# Supplementary material for: Active site specificity profiling datasets of matrix metalloproteinases (MMPs) 1, 2, 3, 7, 8, 9, 12, 13 and 14
Source: Data Brief. 2016 Feb 22;7:299–310. doi: 10.1016/j.dib.2016.02.036 (PMC4777984; doi:10.1016/j.dib.2016.02.036)
Supplement: Supplementary file 10 — Supplementary material [file mmc10.zip › WebPICS_hMMP2_G_1%/P3prime.html]

 

PICS results


|  |  |
| --- | --- |
| **P3prime\_G**  37 in 246 sites   15.0 %    effects > 10 perc. pnts.   (vice-versa in brackets)  P1prime\_I: 14.0 (16.2) |  |
| **P3prime\_K**  18 in 246 sites   7.3 %    effects > 10 perc. pnts.   (vice-versa in brackets)  P3\_V: 32.6 (20.3)   P1\_H: 13.9 (35.6)   P2prime\_K: 20.7 (12.1) |  |
| **P3prime\_N**  18 in 246 sites   7.3 %    effects > 10 perc. pnts.   (vice-versa in brackets)  P1\_D: 13.3 (10.9)   P2prime\_I: 30.2 (15.6) |  |
| **P3prime\_S**  38 in 246 sites   15.4 %    effects > 10 perc. pnts.   (vice-versa in brackets)  P3\_L: 13.1 (19.2) |  |
| **P3prime\_V**  27 in 246 sites   11.0 %    effects > 10 perc. pnts.   (vice-versa in brackets)  P3\_I: 23.6 (19.3)   P1\_G: 25.7 (18.7)   P2prime\_N: 29.2 (79.0) |  |
